# Hydrometallurgical recovery of high-purity molybdenum from spent iron-molybdate catalysts via ammoniacal leaching and anti-solvent crystallization

**DOI:** 10.1038/s41598-026-47825-8

**Published:** 2026-04-09

**Authors:** Muhammad Farhan, Rajiv Ranjan Srivastava, Sadia Ilyas

**Affiliations:** 1 Process Metallurgy, Minerals and Metallurgical Engineering Division, Department of Civil, Environmental and Natural Resources Engineering, Luleå University of Technology, Luleå, 97187 Sweden; 2 Wallenberg Initiative Materials Science for Sustainability (WISE), Department of Civil, Environmental and Natural Resources Engineering, Luleå University of Technology, Luleå, 97187 Sweden

**Keywords:** Spent catalysts, Ammonia-ammonium leaching, Metal recycling, Ammonium heptamolybdate, Quasi-equilibrium of iron-ammine, Chemistry, Engineering, Environmental sciences, Materials science

## Abstract

Molybdenum is a critical raw material for advanced alloys and the chemical industry; however, its supply is increasingly constrained by geopolitical and resource limitations. An efficient recycling of molybdenum from secondary resources is therefore essential to mitigate the supply risk and promote maximum utilization of resources. In this study, a low-energy hydrometallurgical process is proposed for the recovery of molybdenum from spent iron-molybdate catalysts, integrating ammoniacal leaching with anti-solvent crystallization. Conventional leaching using ammonia solution alone achieved less than 90% efficiency and required a high ammonia concentration of 5.0 mol/L. This consumption was significantly reduced to only 2.0 mol/L by employing an ammonia-ammonium solution. The required NH_4_OH-(NH_4_)_2_SO_4_ ratio was optimized to achieve selective molybdenum dissolution that resulted in 92% leaching efficiency while limiting iron co-dissolution to below 5%. The residual iron, governed by the quasi-equilibrium of iron pentaamine complexation, was effectively supressed through controlled settling of the leachate. Subsequently, ethanol-assisted anti-solvent crystallization, performed at an aqueous-to-organic volumetric ratio of 1:2, enabled the direct recovery of high-purity (NH_4_)_6_Mo_7_O_24_(H_2_O)_4_ with 95% yield under ambient conditions. This work demonstrates a scalable and resource-efficient strategy for molybdenum recovery through integrating the selective leaching approach with low-energy crystallization technique.

## Introduction

Molybdenum (Mo) is an important transition metal widely used in the production of structural steels, superalloys, and specific chemicals^1,2^. Driven by its broad industrial relevance, global molybdenum production reached approximately 640 million pounds in 2024, with Europe alone accounting for nearly one-fifth of total consumption^3^. Traditionally, European demand is largely met through imports, primarily from China and Russia^4^. However, recent geopolitical disruption and supply-chain vulnerabilities have significantly challenged this reliance, pushing to identify alternatives and resilient secondary supply routes for this strategically important metal^5,6^.

Recycling the Mo-bearing waste materials significantly offers to mitigate supply-risks while reducing environmental burdens associated with primary mining. Among such resources, spent industrial catalysts represent a particularly attractive reservoir due to high contents of valuable metals therein^7^. Numerous studies have investigated the recovery of molybdenum from spent catalysts using smelting^8^, aqueous dissolution^9,10^, and microbial extraction processes^7–12^. Despite these efforts, relatively few studies have focused on iron-molybdate catalysts, albeit they often contain exceptionally high molybdenum contents (i.e., above 50 wt%). Notably, iron–molybdate catalysts are advantageously employed in formaldehyde synthesis at 300–400 °C, replacing silver-based catalysts that require significantly higher operating temperatures (~ 650 °C). Recycling such catalysts is advantageous not only from an economic and operational perspective but also in terms of reducing the stockpiles of hazardous waste^13,14^.

Hydrometallurgical processing using ammonia leaching has emerged as a preferred approach for iron-molybdate catalysts because molybdenum readily forms soluble molybdate species under alkaline conditions, while keeping iron in residues^15–17^. Babichev et al.^15^ reported about 95% molybdenum leaching using ammonia concentrations four times higher than stoichiometric requirements at elevated temperature (80 °C). In a different study, Qi et al.^16^ achieved comparable leaching efficiencies at room temperature using 2.6 mol/L ammonia; however, they subsequently tested hydrothermal precipitation to prepare precursor for reuse in same type of catalysts. They did not address any purification and recovery steps for molybdate solution suitable for broader industrial reuse. In contrast, soda-roasting at 771 °C has been explored by He et al.^17^ to achieve 92.2% molybdenum dissolution, while Mohammadi et al.^14^ demonstrated nearly complete molybdenum leaching through direct leaching followed by zinc cementation and calcination to produce MoO_3_ of high purity. Despite effective recovery with desirable purity, these approaches involve high energy inputs, additional reagents, and secondary metal contamination, pressing the need of further purification steps and increasing processing costs. Consequently, a simple and efficient process is required to overcome these issues with a hydrometallurgical operation.

In this context, the present study was aimed to investigate an ammoniacal leaching system incorporating ammonia-ammonium salt mixtures to reduce total ammonia consumption while maintaining high molybdenum recovery. Furthermore, an anti-solvent crystallization (ASC) strategy is integrated for molybdenum recovery, enabling low-energy precipitation of high-purity molybdate product. To the best of our knowledge, the application of ASC for molybdenum recovery from any spent catalysts has not been reported yet. Notably, ASC is a simple and effective approach to recover metal salts by adding a water-miscible organic solvent with a lower dielectric constant than water/aqueous media^18^. The addition of such organic disrupts metal ion solvation and results in a reduced solubility of the dissolved metal ions^19^. Importantly, the volatility of commonly used organic anti-solvents and the established recyclability of free ammonia offer additional opportunities for process integration and sustainability^20,21^; however, this was not in focus of the study and will be optimized later during the monitoring of closed-loop operation.

## Experimental

### Materials

**Fig. 1:**
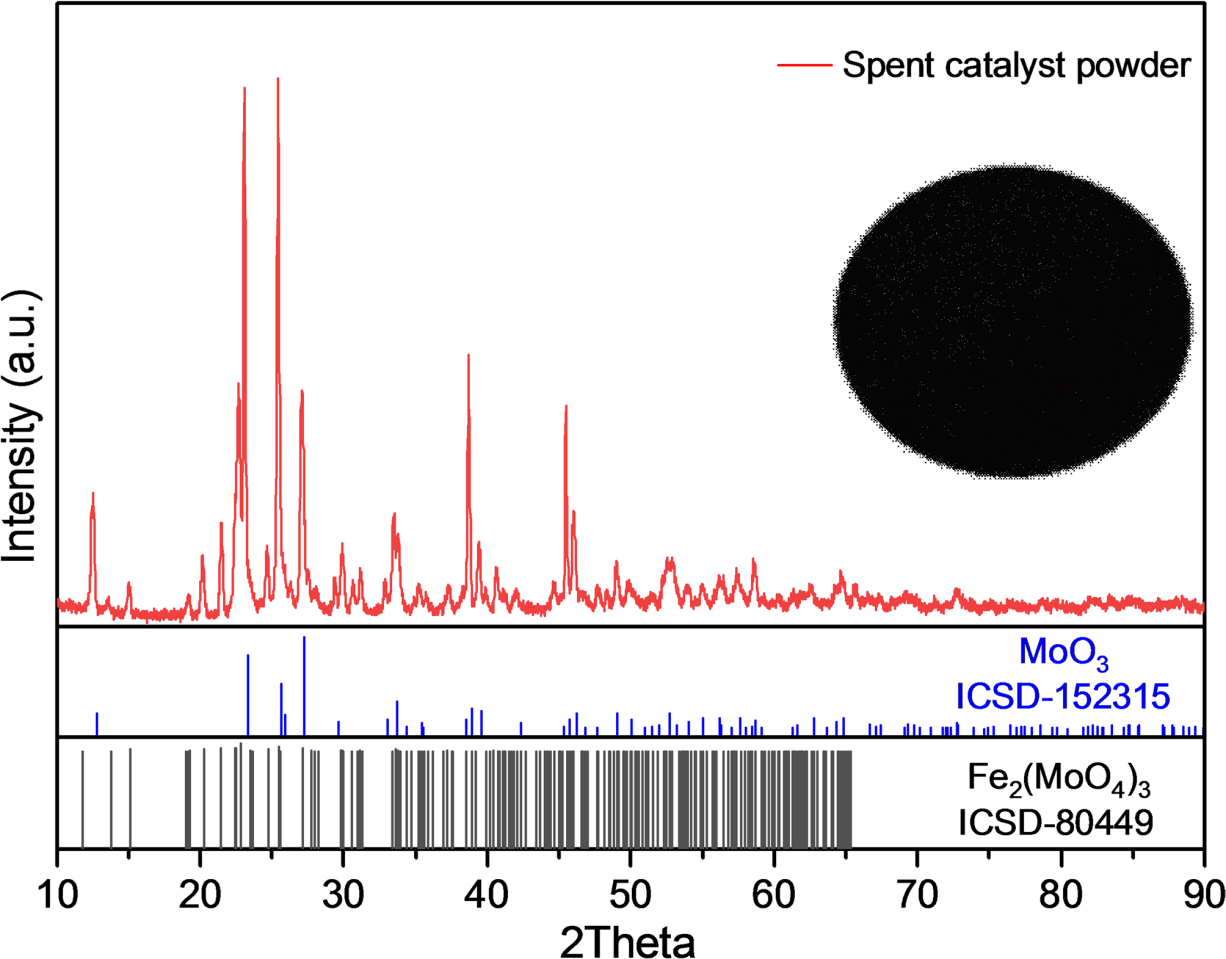
The pattern acquired by performing the powder XRD of spent iron-molybdate catalyst used in this study.

Dark grey cylindrical pellets of spent iron-molybdate catalysts were obtained from a chemical industry end-user. The material was crushed and finely ground using a mortar and pestle, followed by sieving to obtain a size fraction of − 200 mesh to be used for analysis and leaching studies. For elemental characterization, 1.0 g of ground sample was digested in 100 mL of freshly prepared aqua regia under boiling conditions to ensure complete dissolution. The resulting solution was filtered in hot condition, and the residue was thoroughly washed with hot deionized water Milli-Q water (conductivity 0.055 µS/cm, resistivity 18.2 MΩ-cm). The combined filtrate and washings were collected in a 100 mL volumetric flask wherein volume make up was done using 5 vol% HNO_3_ solution. An appropriate aliquot of this solution was then further diluted with 2 vol% HNO_3_ prior to analyze metal concentration using inductively coupled plasma optical emission spectroscopy (ICP-OES; iCAP 7000 series, Thermo Fisher Scientific, USA). Based on the analytical results for digestions performed in duplicates, the spent catalyst contained 52.59 wt% molybdenum, 12,98 wt% iron, and 0.07 wt% aluminum (Table 1). Phase identification of the sample was carried out using powder X-ray diffractometer (XRD; Malvern Panalytical Aeris: Cu Kα radiation, 45 kV, 40 mA), which revealed major peaks corresponding to Fe_2_(MoO_4_)_3_ and MoO_3_ (Fig. 1). All reagents used in this study were of analytical grade, including ammonium hydroxide (NH_4_OH, 28 wt%, VWR International), ammonium sulfate (NH_4_)_2_SO_4_, ≥ 99.5 wt%, Merck), hydrochloric acid (HCl ≥ 36 wt%, Fisher scientific), nitric acid (HNO_3_, ≥ 68 wt%, Merck), and ethanol (C_2_H_5_OH, Fisher scientific).

**Table 1:** Elemental analysis of the spent iron molybdate catalysts sample used in this study.

Elements	Molybdenum	Iron	Aluminium
Wt.%	52.59 ± 0.80	12.98 ± 0.43	0.07 ± 0.03

### Leaching procedures

All the leaching experiments were conducted in using a LabTech Xelsius reaction station (LabTech Srl, Italy) equipped with an automated reaction controller, ten reaction cells, and an integrated water-circulation temperature control system. For each experiment, 1.5 g of sample was leached in 15 mL of freshly prepared ammonia/cal solution (to maintain a fixed pulp density of 10%) with ammonia concentration ranging from 0 to 5 mol/L, using 30 mL capped glass vials placed within the reaction cells. To prepare a buffer leaching solution, a predetermined weight of ammonium sulfate was primarily dissolved in a calculated volume of ammonium hydroxide, followed by dilution with Milli-Q water to achieve a desired concentration of total NH_3_ in the solution. The experimental conditions for each reaction cell were individually controlled and maintained at a pre-determined leaching time of 60 min, temperature of 20 °C, and stirring speed of 300 rpm. An Eh-pH meter (Thermo-Fisher) was separately inserted to take the initial and final readings of leaching solution.

Soon after the completion of the leaching time, the slurry was filtered using a Buckner funnel under vacuum to achieve solid-liquid separation. The solid residue was subsequently washed with 5 (vol%) NH_4_OH solution, and the washings were combined with the leached filtrate prior to metal concentration analysis. The solution thus collected was appropriately diluted with 2 vol% HNO_3_ and analyzed using ICP-OES. The obtained values of metal concentrations were used to calculate the leaching efficiency as follows:

1 $$\:\mathrm{L}\mathrm{e}\mathrm{a}\mathrm{c}\mathrm{h}\mathrm{i}\mathrm{n}\mathrm{g}\:\left({\%}\right)\:=\:\frac{{C}_{LL\:}\times\:\:{V}_{LL}}{Q\:\times\:\:\propto\:}\:\times\:100$$

where, *C*_*LL*_ = metal concentration in leach liquor (g/L), *V*_*LL*_ = volume of leach liquor + wash liquor (L), *Q* = feed mass of the sample (g), and *α* = mass fraction of metal in the feed sample (%). Each leaching condition was tested in duplicate, and the reported results represent the mean values, with error bars indicating the deviation between repeated experiments. The solid residues were dried overnight at 50 °C before performing the characterization using powder XRD.

### Recovery procedures

A total of 500 mL leach liquor was generated using an ammoniacal solution with total NH_3_ concentration 2.0 mol/L at NH_4_OH-(NH_4_)_2_SO_4_ molar ratio equal to 3:1. The resulting leachate was settled for 12 h to allow iron precipitation from the ammoniacal solution. The supernatant was subsequently filtered to obtain an iron-depleted solution, which contained 48.2 g/L molybdenum and < 7 mg/L iron (Eh, − 106 mV and pH, 10.32). This solution was used for subsequent ASC studies. All the experiments were conducted in reaction vials placed within the LabTech Xelsius reaction station (LabTech Srl, Italy) using a fixed total volume of 12 mL at different aqueous-to-organic (A/O) volumetric ratio (ranging from 0.5 to 2). All ASC experiments were performed at a constant temperature (20 °C) and the antisolvent organic was added at once at zero time. Further, molybdenum recovery efficiency was examined at varied stirring speed (200–600 rpm) and mixing time (10–60 min), as individually set for each reaction cell. After the completion of ASC duration, stirring were stopped and the suspension was allowed to settle for 30 min. The resulting crystals were then separated using a Buckner funnel, and the collected mother liquor was diluted and analyzed to determine molybdenum recovery efficiency as follows:

2 $$\:\mathrm{R}\mathrm{e}\mathrm{c}\mathrm{o}\mathrm{v}\mathrm{e}\mathrm{r}\mathrm{y}\:\left({\%}\right)\:=\frac{\left({C}_{iL\:}\times\:\:{V}_{iL}\right)-\left({C}_{mL\:}\times\:\:{V}_{mL}\right)}{\left({C}_{iL\:}\times\:\:{V}_{iL}\right)}\:\times\:100$$

where, *C*_*iL*_ = metal concentration in initial solution (g/L), *V*_*iL*_ = volume of initial solution (L), *C*_*mL*_ = metal concentration in mother liquor (g/L), *V*_*mL*_ = remained volume of the mother liquor (L). Each ASC experimental condition was tested in duplicate, and the results are presented as mean values with standard error. The crystals collected were separately washed with 5 mL ethanol and then dried overnight at 30 °C before analyses using the SEM-EDX and powder XRD.

## Results

### Leaching in ammonia solution as a function of NH_3_ concentration

**Fig. 2:**
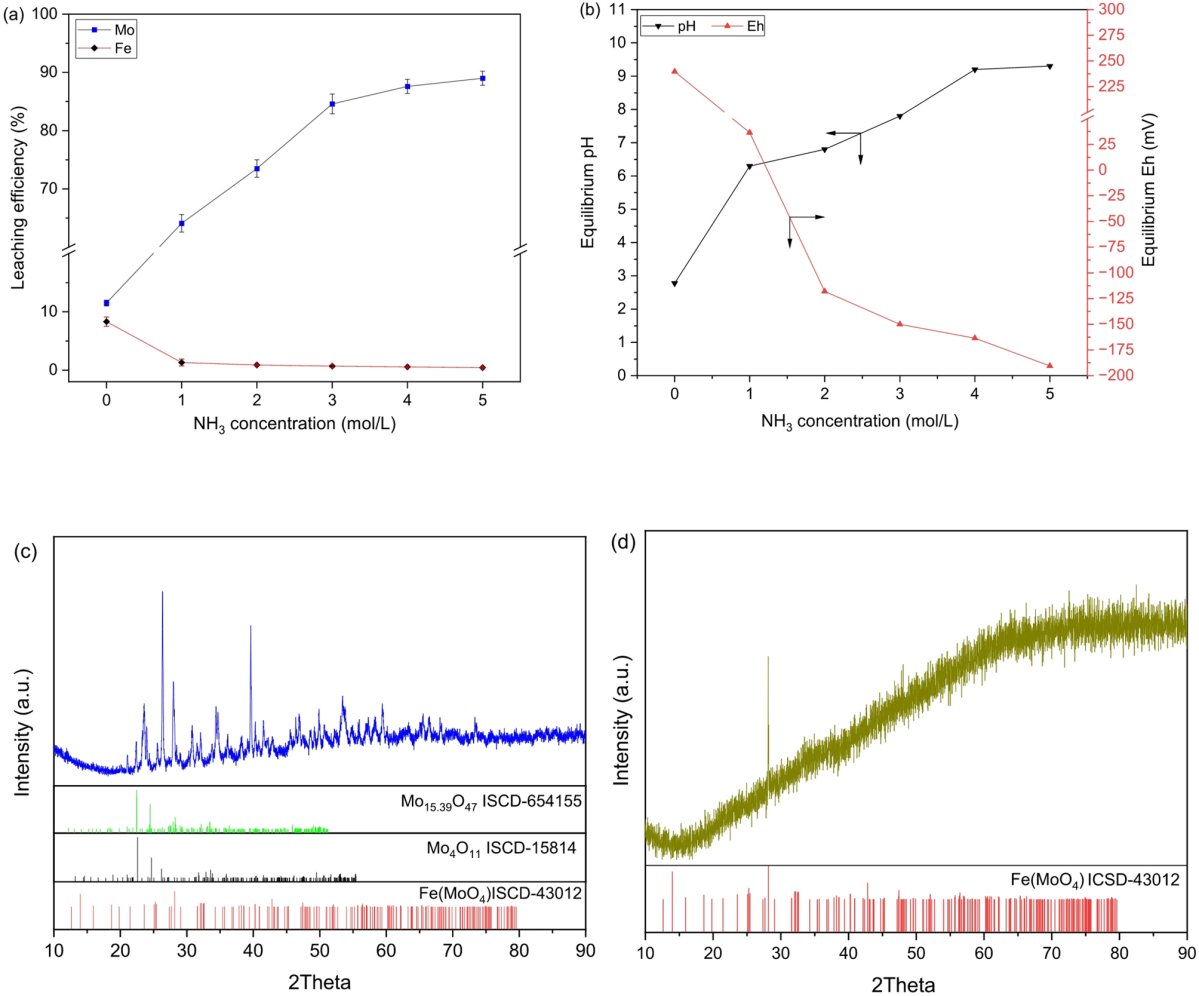
(**a**) Effect of NH_3_ concentration on leaching efficiency of metals from spent catalysts, and (**b**) the corresponding equilibrium pH and Eh of the leached solutions. The pattern acquired by performing the powder XRD of residues obtained after (**c**) water and (**d**) 3.0 mol/L ammonia leaching. (experimental condition: pulp density = 10%, temperature = 20 °C, stirring speed = 300 rpm, and time = 60 min).

The leaching behavior of metals (molybdenum and iron) in ammonia solution was investigated by varying the ammonia concentration from 0 to 5 mol/L, while maintaining all other parameters invariable (i.e., pulp density = 10%, temperature = 20 °C, stirring speed = 300 rpm, and time = 60 min). Figure 2 illustrates the leaching efficiencies of metals along with the corresponding Eh and pH values across different ammonia concentrations. In deionized water alone, approximately 11% molybdenum and ~ 8% of iron could be leached with solution Eh, 239.6 mV and pH, 2.78. The powder XRD of water-leached residue (Fig. 2c) also showed the persistence of molybdate phases (i.e., Fe(MoO_4_), Mo_4_O_11_, and Mo_15.39_O_47_), indicating minimal breakdown of iron-molybdate matrices in water alone. In contrast, the introduction of ammonia markedly altered the leaching behavior. Iron dissolution was strongly supressed to below 1.5%, whereas molybdenum dissolution increased sharply with increasing NH_3_ concentrations (rising from 64% to 89% using 1 to 5 mol/L NH_3_). This leaching trend indicates that under increasingly alkaline condition, molybdenum preferentially forms soluble species, while iron undergoes hydrolysis and remains unleached in residue. Notably, molybdenum leaching exhibited only marginal improvement beyond a 3 mol/L NH_3_ solution (Fig. 2a), suggesting that further increases in alkalinity do not significantly enhance the leaching driving force under the investigated conditions. XRD analysis of the ammonia-leached residue (Fig. 2d) revealed a predominantly non-crystalline phase, consistent with the formation of amorphous iron hydroxides, while a major residual peak at 28.12° corresponds to Fe(MoO_4_), indicating incomplete lattice breakdown at higher ammonia concentrations.

### Effect of ammonia-ammonium system

**Fig. 3:**
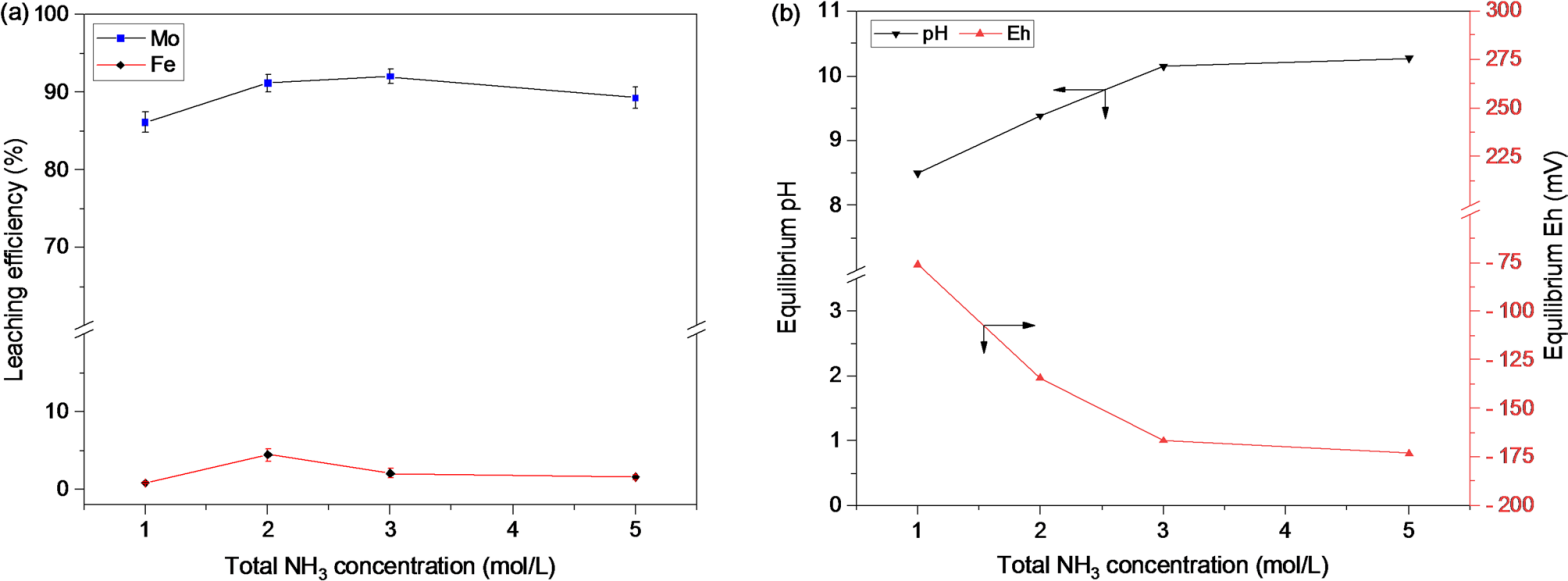
(**a**) Effect of total NH_3_ concentration on metal leaching while contribution from NH_4_OH-(NH_4_)_2_SO_4_ comes at 1:1 at each stage, and (**b**) corresponding equilibrium pH and Eh of the leached solutions (condition: pulp density = 10%, temperature = 20 °C, stirring speed = 300 rpm, and time = 60 min).

To further improve molybdenum leaching by providing a more favorable alkaline environment, the influence of ammonia-ammonium buffer system was investigated. The total ammonia concentration was varied between 1.0 and 5.0 mol/L while maintaining an equimolar ratio of ammonia and ammonium salt. As shown in Fig. 3a, the introduction of the buffer system exerted a pronounced effect on molybdenum dissolution, reaching above 91% leaching efficiency at a total NH_3_ concentration of 2 mol/L only. This improvement can be attributed to the shift in solution chemistry of the ammoniacal system, particularly the buffering effect exhibited above the pKa value 9.25. The results showed that at total NH_3_ concentration ≥ 2.0 mol/L, the equilibrium pH increased to ≥ 9.4, while the Eh value decreased below − 134 mV. In comparison, at 1.0 mol/L total NH_3_ and ammonia-ammonium molar ratio = 1:1, the equilibrium pH and Eh were 8.5 and − 76 mV, respectively (Fig. 3b).

In contrast, when using NH_4_OH solution alone at the same ammonia concentrations, the equilibrium pH was significantly lower (refer to Fig. 2b). This highlights the critical role of the ammonia-ammonium buffer in maintaining alkaline conditions conducive to molybdenum dissolution. A minor co-dissolution of iron (~ 4.5%) was observed at 2.0 mol/L total NH_3_, likely due to the formation of iron-ammine complexes^22^, despite iron generally exhibiting limited solubility under alkaline conditions. Furthermore, a favorable dissolution condition (i.e., equilibrium pH = 10.27 and Eh = − 173 mV) using a higher ammonia solution (total NH_3_ concentration = 5.0 mol/L) was provided; however, a decline in molybdenum leaching was observed. This behavior can be attributed to the common-ion effect associated with increased sulfate concentrations in the buffer system^23^, which may inhibit molybdenum dissolution and/or promote partial re-adsorption of molybdenum species onto solid surfaces. Based on these observations, the role of ammonia-ammonium ratio was studied in the next set of experiment.

### Effect of NH_3_-NH_4_^+^ ratio in the ammoniacal system

**Fig. 4:**
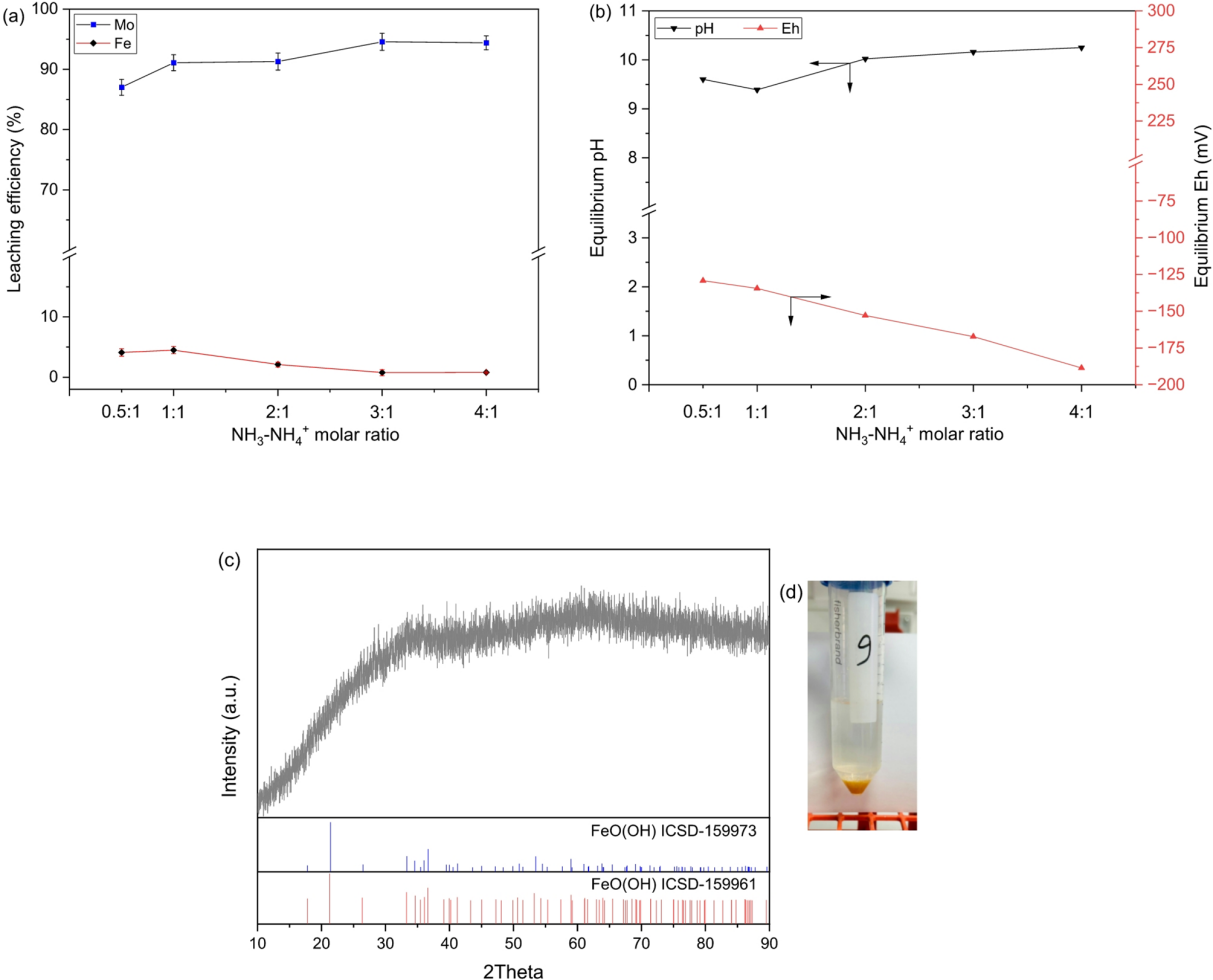
(**a**) Effect of NH_4_OH-(NH_4_)_2_SO_4_ ratio on metals leaching from spent iron-molybdate catalyst at total ammonia concentration of 2 mol/L along with pulp density of 10%, 300 rpm, room temperature, and 60 min, (**b**) corresponding equilibrium pH and Eh of the leached solutions. (**c**) The pattern acquired by performing the powder XRD of the residue obtained by leaching with ammoniacal solution wherein total NH_3_ concentration = 2 mol/L at NH_4_OH-(NH_4_)_2_SO_4_ ratio of 3:1. (**d**) Settling of iron precipitates observed after filtration in the ammoniacal leached solution.

The effect of ammonia-ammonium salt ratio within the ammoniacal buffer system was subsequently investigated by varying molar contribution ratio from both ammonia and ammonium salt within 0.5 to 4, while keeping total NH_3_ concentration unchanged at 2 mol/L in the solution. Figure 4a shows that molybdenum leaching was greatly affected by the change in ammonium salt concentration in the solution. A lower ratio of NH_3_:NH_4_^+^ at 0.5:1 limits molybdenum leaching to 87% only. The leaching significantly increased up to ~ 95% with lowering salt concentration contributing to total ammonia concentration at a ratio of ≥ 3:1. This trend further supports the occurrence of common-ion effect at higher salt concentrations, which supress molybdenum dissolution^23^. In contrast, iron leaching decreased with increasing contribution from ammonia solution, which can be ascribed through the Eh-pH of the system. As can be seen from Fig. 4b, increasing the NH_3_:NH_4_^+^ ratio shifted the solution toward more negative Eh within a pH range of 9.6 to 10.25, thereby favoring precipitation of iron hydroxide^22^. This is further supported by the amorphous texture of the residue and the corresponding XRD patterns (Fig. 4c). Interestingly, sample kept for a long duration showed iron settling at the bottom of the tubes (Fig. 4d), which confirms that iron dissolution in the ammoniacal system occurs under quasi-equilibrium that gradually shifts to stable hydroxide formation^24^.

### Molybdenum recovery via anti-solvent crystallization

**Fig. 5:**
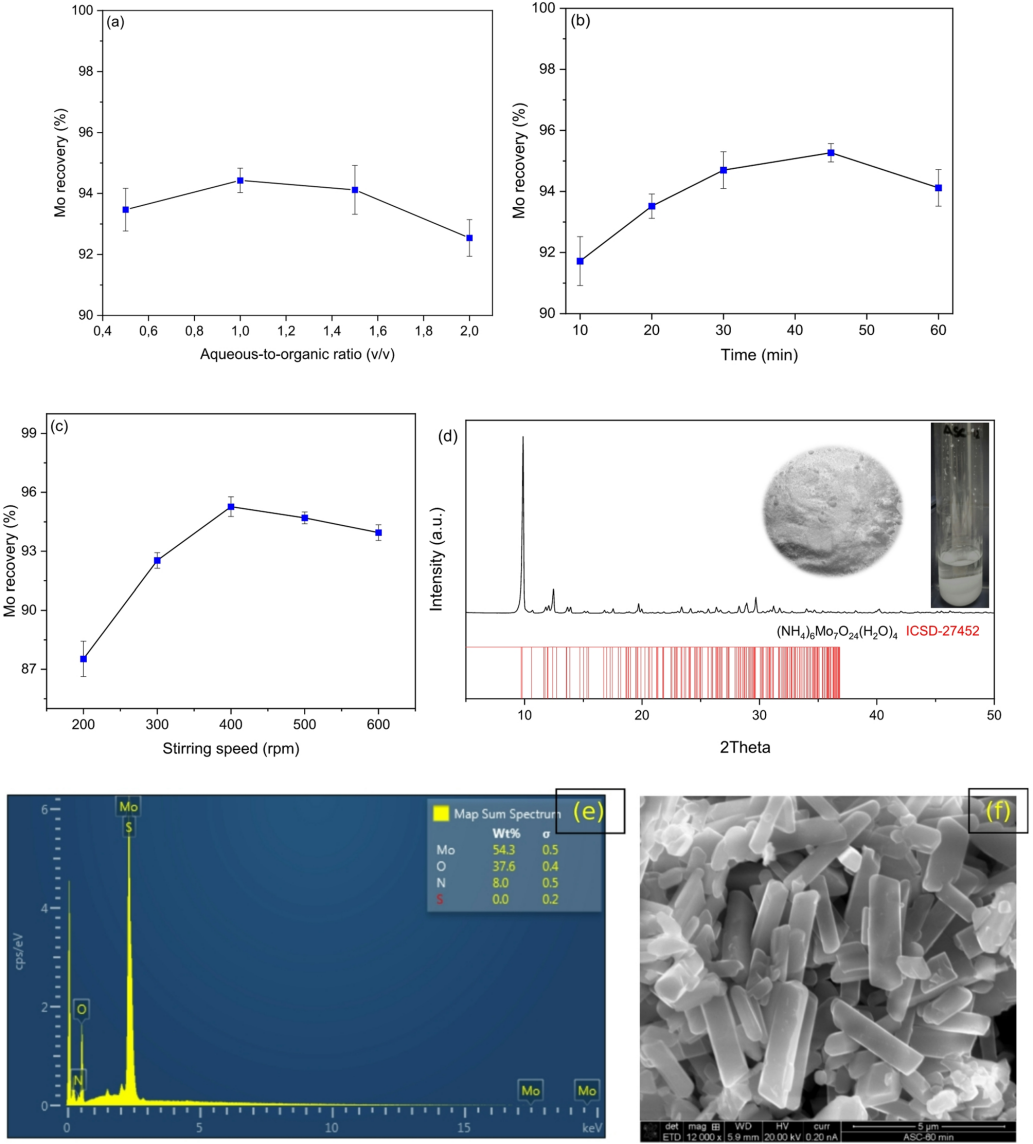
Anti-solvent crystallization of molybdenum from ammoniacal leach liquor by mixing ethanol as a function of (**a**) aqueous-to-organic (A/O) ratio at stirring speed, 400 rpm; mixing time, 60 min; settling time, 30 min; (**b**) stirring time at A/O ratio, 1.5; stirring speed, 400 rpm; settling time, 30 min; and (**c**) stirring speed at A/O ratio, 1.5; mixing time, 45 min; settling time, 30 min. (**d**) The pattern acquired by performing the powder XRD and (**e**) SEM-EDX of the precipitated salt after anti-solvent crystallization at the optimal condition (i.e., A/O = 1.5, stirring speed = 400 rpm, mixing time = 45 min, settling time = 30 min).

The iron-depleted molybdate solution (as described in Sect. 2.3) was used for the ASC studies. Ethanol was selected as the organic precipitant due to its lower dielectric constant (16.2) compared with that of the ammonia solution (31.6)^25^. The addition of ethanol into ammoniacal solution disrupts the hydration shell surrounding dissolved metal ions^19,21^, thereby reducing the solubility of molybdate species and promoting their precipitation. To achieve sufficient disruption of the solvation environment, the amount of organic precipitant was optimized by varying the A/O volumetric ratio from 0.5 to 2. Other parameters like stirring speed (400 rpm), mixing time (60 min), and settling time (30 min) were kept invariable. Results in Fig. 5a depict high yields of molybdenum precipitation, over 92% at a maximum A/O volumetric ratio of 2. Although this efficiency was slightly lower than that of the precipitation obtained at an A/O of 1 (94.4%), an A/O ratio = 2 was optimized for the next studies on mixing time variation due to less requirement of organic precipitant.

Figure 5b reveals that the mixing time between two miscible solutions has certain effect on molybdenum recovery, and the precipitation efficiency reached a maximum of 95.3% at 45 min. Beyond this point, a slight decline in precipitation was observed. This behavior can be attributed to partial re-dissolution of the precipitates caused by prolonged agitation. Extended mixing may lead to crystal breakage, thereby increasing the surface area-to-volume ratio and promotes the re-dissolution of molybdate species into the ammoniacal solution. This behavior is further supported by the results shown in Fig. 5c, where an increase in stirring speed (> 400 rpm) resulted in decreased molybdenum precipitation efficiency. A higher agitation reduces the thickness of the Nernst boundary layer, thereby enhancing mass transfer of dissolved species away from the solid surface and promoting re-dissolution of smaller particles^26,27^. On contrary, at lower stirring speeds (≤ 300 rpm), reduced mixing intensity may require longer mixing times (> 45 min) to reach the maximum precipitation efficiency.

## Discussion

The leaching of spent iron molybdate catalysts, primarily composed of MoO_3_ and Fe_2_(MoO_4_)_3_, in ammonia solution are commonly driven via the following reactions^16^:

3 $$\:\mathrm{M}\mathrm{o}{\mathrm{O}}_{3}+2\mathrm{N}{\mathrm{H}}_{3}\cdot {\mathrm{H}}_{2}\mathrm{O}\:\to\:\:(\mathrm{N}{\mathrm{H}}_{4}{)}_{2}\mathrm{M}\mathrm{o}{\mathrm{O}}_{4}+\:{\mathrm{H}}_{2}\mathrm{O}\:$$

4 $$\:{\mathrm{F}\mathrm{e}}_{2}(\mathrm{M}\mathrm{o}{\mathrm{O}}_{4}{)}_{3}+6\mathrm{N}{\mathrm{H}}_{3}\cdot {\mathrm{H}}_{2}\mathrm{O}\:\to\:\:3(\mathrm{N}{\mathrm{H}}_{4}{)}_{2}\mathrm{M}\mathrm{o}{\mathrm{O}}_{4}+2\mathrm{F}\mathrm{e}(\mathrm{O}\mathrm{H}{)}_{3}\downarrow\:\:$$

Although leaching in 5.0 mol/L ammonia solution achieved 89% efficiency, the associated ammonia consumption was very high. This issue was addressed by employing an ammoniacal buffer system of NH_4_OH-(NH_4_)_2_SO_4_, which enabled higher molybdenum recovery (~ 92%) at a substantially lower total NH_3_ concentration (i.e., 2.0 mol/L). This approach resulted in a significant reduction in usage of ammonia solution, corresponding to a decrease in NH_4_OH consumption to approximately 212.5 mL per litre of the alkaline lixiviant. In terms of total ammonia consumption, this corresponds to 1.82 g NH_3_ per gram of molybdenum leached using NH_4_OH alone, compared to only 0.7 g NH_3_ per gram of molybdenum when using the NH_4_OH-(NH_4_)_2_SO_4_ system. The enhanced leaching performance in the buffered ammoniacal system can be attributed to the following reactions:

5 $$\:\mathrm{N}{\mathrm{H}}_{3}\cdot {\mathrm{H}}_{2}\mathrm{O}\:\leftrightarrow\:\:\mathrm{N}{\mathrm{H}}_{4}^{+}+\mathrm{O}{\mathrm{H}}^{-}$$

6 $$\:(\mathrm{N}{\mathrm{H}}_{4}{)}_{2}\mathrm{S}{\mathrm{O}}_{4}+\:{\mathrm{H}}_{2}\mathrm{O}\:\to\:\:2\mathrm{N}{\mathrm{H}}_{4}^{+}+\mathrm{S}{\mathrm{O}}_{4}^{2-}$$

7 $$\:2\mathrm{N}{\mathrm{H}}_{3}.{\mathrm{H}}_{2}\mathrm{O}+\:(\mathrm{N}{\mathrm{H}}_{4}{)}_{2}\mathrm{S}{\mathrm{O}}_{4}\to\:\:3\mathrm{N}{\mathrm{H}}_{4}^{+}+\mathrm{O}{\mathrm{H}}^{-}+\mathrm{S}{\mathrm{O}}_{4}^{2-}$$

8 $$\:{\mathrm{F}\mathrm{e}}_{2}(\mathrm{M}\mathrm{o}{\mathrm{O}}_{4}{)}_{3}+6\mathrm{N}{\mathrm{H}}_{4}^{+}+6\mathrm{O}{\mathrm{H}}^{-}+\:\mathrm{S}{\mathrm{O}}_{4}^{2-}\to\:\:3(\mathrm{N}{\mathrm{H}}_{4}{)}_{2}\mathrm{M}\mathrm{o}{\mathrm{O}}_{4}+2\mathrm{F}\mathrm{e}(\mathrm{O}\mathrm{H}{)}_{3}\downarrow\:+\:\mathrm{S}{\mathrm{O}}_{4}^{2-}\:$$

9 $$\:\mathrm{M}\mathrm{o}{\mathrm{O}}_{3}+2\mathrm{N}{\mathrm{H}}_{4}^{+}+2\mathrm{O}{\mathrm{H}}^{-}\:\to\:\:(\mathrm{N}{\mathrm{H}}_{4}{)}_{2}\mathrm{M}\mathrm{o}{\mathrm{O}}_{4}+\:{\mathrm{H}}_{2}\mathrm{O}\:$$

However, a prolonged operation of iron molybdate catalysts tends to degrade and forming a fraction of FeMoO_4_, in which iron exists in the + 2 state^28,29^. This exhibits distinct leaching behavior compared to Fe_2_(MoO_4_)_3_, and its dissolution in the ammoniacal system can be written as follows:

10 $$\:3\mathrm{F}\mathrm{e}\mathrm{M}\mathrm{o}{\mathrm{O}}_{4}+6\mathrm{N}{\mathrm{H}}_{4}^{+}+6\mathrm{O}{\mathrm{H}}^{-}+\:\mathrm{S}{\mathrm{O}}_{4}^{2-}\to\:\:3(\mathrm{N}{\mathrm{H}}_{4}{)}_{2}\mathrm{M}\mathrm{o}{\mathrm{O}}_{4}+3\mathrm{F}\mathrm{e}(\mathrm{O}\mathrm{H}{)}_{2}\downarrow\:+\:\mathrm{S}{\mathrm{O}}_{4}^{2-}\:$$

11 $$\:3\mathrm{F}\mathrm{e}(\mathrm{O}\mathrm{H}{)}_{2}+5\mathrm{N}{\mathrm{H}}_{3}\to\:\:{\mathrm{F}\mathrm{e}\left(\mathrm{N}{\mathrm{H}}_{3}\right)}_{5}^{2+}+2\mathrm{O}{\mathrm{H}}^{-}\:$$

Accordingly, the partial dissolution of iron observed in this study can be directly correlated to the presence of Fe(II) species, which undergo transient solubilization via the formation of iron(II) pentaamine complex under quasi-equilibrium^22^. The thus formed Fe(NH_3_)_5_^2+^ gradually undergoes ligand exchange in the presence of hydroxyl ions that tends to the precipitation of iron hydroxide upon extended aging of the solution^24^.

For molybdenum recovery purpose, the application of ASC technique using ethanol as the organic precipitant showed promising results. The process exhibited reverse solubility behavior of molybdenum, leading to the formation of (NH_4_)_6_Mo_7_O_24_(H_2_O)_4_ (Fig. 5d) with a purity above 99.9% (Fig. 5e). This behavior can be attributed to the ability of ethanol to reduce the overall polarity and dielectric constant of the ammoniacal solution, thereby weakening molybdate solvation and promoting crystallization^30^. On the other hand, the process advantage remains in the high product yields within a narrow temperature range^31^, with effective crystallization occurring at room temperature (20 °C) in this study. SEM micrograph of the product revealed a uniform distribution of molybdate crystals with rectangular shape and sizes on the order of magnitude 10^–2^ (Fig. 5f). Notably, the ASC technique produced significantly finer crystals, with an average particle size of 3.7 μm, compared to 57.8 μm obtained via conventional evaporation methods^32^. Notably, ammonium heptamolybdate with particle sizes below 5.0 μm is advantageous as a precursor for various industrial applications, including catalysts production, electroplating, foliar fertilizer, and biological imaging^33,34^.

## Conclusions

In summary, this study disclosed a novel, simple, and efficient process for recycling molybdenum from a specific iron-molybdate catalyst waste. The study applied NH_4_OH-(NH_4_)_2_SO_4_ buffer solution to effectively lower down the reagent consumption, achieving over 92% leaching efficiency with only 2.0 mol/L total ammonia concentration. Additionally, the novel application of anti-solvent crystallization in ammoniacal molybdate solution using ethanol enabled > 95% molybdenum recovery as (NH_4_)_6_Mo_7_O_24_(H_2_O)_4_ with a product purity over 99.9%. A key advantage of this process is the elimination of external heating, as all unit operations were conducted at ambient temperature, highlighting its energy efficiency. Overall, this work demonstrates a technically feasible approach for molybdenum recovery from spent catalysts and offers a sustainable strategy to alleviate the growing supply-risk of this strategic metal.

## Data Availability

The data is protected as per the Swedish law. Any request with reasonable cause should come through a proper channel.
